# Immediate-Onset Acute Pancreatitis During Transarterial Chemoembolization: A Case Report

**DOI:** 10.7759/cureus.96400

**Published:** 2025-11-09

**Authors:** Yuan Shen, Xuejie Deng, Yuqing Chen, Jiaming Lei

**Affiliations:** 1 Department of Gastroenterology, People’s Hospital of Leshan, Leshan, CHN

**Keywords:** acute pancreatitis, embolization, hepatocellular carcinoma, liver cirrhosis, transarterial chemoembolization

## Abstract

Transarterial chemoembolization (TACE) is widely used in treating unresectable hepatocellular carcinoma (HCC), while acute pancreatitis represents a rare but serious complication of this procedure. This article reports the case of a 57-year-old male with hepatitis B-related liver cirrhosis and China Liver Cancer stage IIb HCC who experienced the abrupt onset of acute pancreatitis during the TACE procedure, immediately following the embolic infusion. By reviewing the patient's clinical presentation, imaging data, laboratory findings, and treatment process, we infer that the acute pancreatitis was most likely caused by the reflux of embolic material, leading to pancreatic ischemia. This report focuses on the clinical features, underlying mechanisms, and prevention strategies of acute pancreatitis complicating TACE procedures.

## Introduction

Primary liver cancer, particularly hepatocellular carcinoma (HCC), ranks among the most prevalent and lethal malignancies worldwide. Recent epidemiological data indicate persistently high annual incidence rates, posing a significant burden on global public health systems [1]. Among various treatment modalities for HCC, transarterial chemoembolization (TACE) has become an indispensable locoregional therapy for patients with intermediate-stage disease, playing a pivotal role in clinical practice. Current data indicate that a substantial number of HCC patients worldwide undergo TACE treatment, making a thorough understanding and standardized management of its complications particularly important [2].

TACE, while a targeted therapy, carries an inherent risk of non-target embolization. Its precision is not absolute and is highly dependent on factors such as vascular anatomy, catheter tip position, and injection technique. This limitation is central to the pathophysiology of the complication described in our case. However, while providing therapeutic benefits, this procedure carries risks of various complications. Beyond the common post-embolization syndrome (PES), it can also cause severe adverse events, including liver function impairment and non-target embolization [2]. Acute pancreatitis (AP) is a common and critical condition in gastroenterology, with an overall incidence that is showing an increasing trend. Once progressing to a severe form, mortality rates increase significantly [3]. Among these, acute pancreatitis, though uncommon, represents a serious complication that demands attention due to its abrupt onset and potentially fatal outcomes [4,5].

This paper reports a case of a 57-year-old male with hepatitis B cirrhosis and China Liver Cancer (CNLC) stage IIb HCC who developed acute pancreatitis during TACE treatment. Through the analysis of this case, which showcases the immediate intra-procedural onset of AP, we aim to elucidate the potential reflux-related mechanism and highlight the diagnostic challenges in distinguishing it from PES. Our objective is to provide specific insights that enhance intraprocedural vigilance and guide post-TACE management.

## Case presentation

A 57-year-old male with a five-year history of hepatitis B-related liver cirrhosis, without regular follow-up, presented with complaints of dull right upper quadrant pain. Physical examination revealed a chronically ill appearance with mild percussion tenderness over the liver region, without significant tenderness in other abdominal areas.

**Figure 1:**
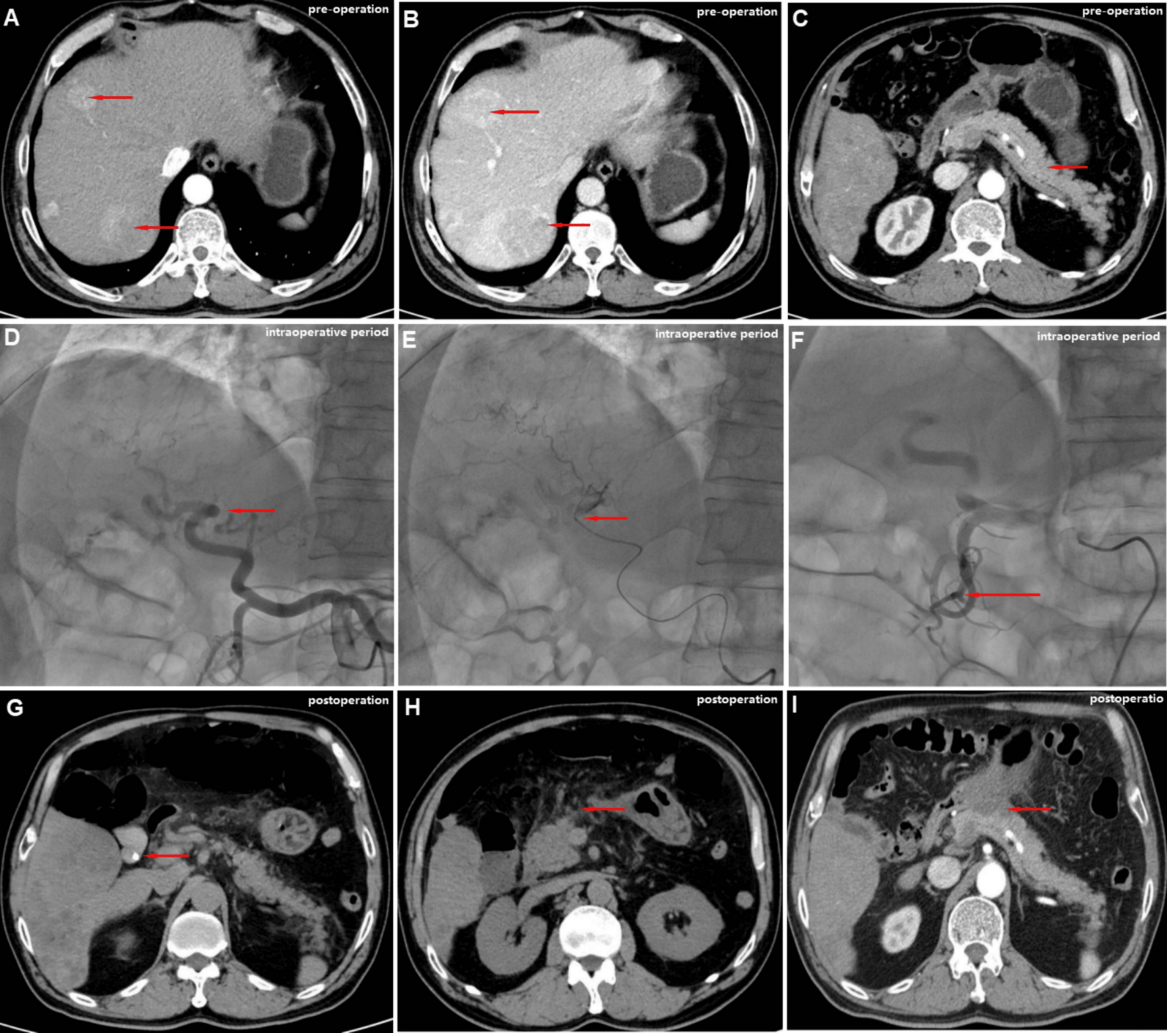
Patient's preoperative contrast-enhanced CT findings of the liver lesions, fluoroscopy-guided TACE procedure, and postoperative CT manifestations of pancreatitis. Scale bar: the red arrow represents 5 cm. (A, B) The red arrows indicate the multiple lesions in the liver. The contrast-enhanced CT demonstrates the classic "wash-in and wash-out" enhancement pattern characteristic of HCC. (C) The red arrow shows the normal appearance of the pancreas prior to the procedure. (D) The red arrow highlights the tortuous, pathological vasculature of the hepatic tumor. (E) The red arrow indicates the microcatheter positioned superselectively in a distal branch for the injection of drug-eluting microspheres. This illustrates the final superselective catheter position prior to embolization. (F) Post-embolization angiography via the RH catheter for evaluation. The angiogram shows decelerated blood flow within the treated vessel. (G) The red arrow points to the gallstone in the gallbladder neck. (H) Postoperative CT showing extensive peripancreatic stranding and fluid collection, indicative of pancreatitis (red arrow). (I) Follow-up contrast-enhanced CT one month later reveals a walled-off necrosis in the pancreatic head (red arrow). CT: computed tomography, TACE: transarterial chemoembolization, HCC: hepatocellular carcinoma

A contrast-enhanced CT scan demonstrated an irregular liver contour with serrated edges and widened hepatic fissures, most notable in the portal venous phase. Within the liver parenchyma, multiple hypodense nodular/mass lesions were identified. The largest lesion, approximately 7.4 cm × 6.9 cm, was located near the inferior border of the right hepatic lobe. It exhibited the classic radiological hallmarks of HCC, including non-rim arterial phase hyperenhancement with subsequent washout in the portal venous and delayed phases, thereby meeting the imaging diagnostic criteria according to the CNLC guidelines (Figure 1A-1B) [1]. On the non-contrast phase, the gallbladder was not distended, but a high-density nodule measuring approximately 0.6 cm was noted in the gallbladder neck. No significant dilation of the intra- or extrahepatic bile ducts was observed. Throughout all phases, the pancreas appeared normal in morphology with homogeneous parenchymal density and clear peripancreatic fat planes (Figure 1C).

Laboratory investigations revealed significantly elevated alpha-fetoprotein levels at 986.8 ng/mL and hepatitis B surface antigen levels greater than 250 IU/mL. Complete blood count, liver and kidney function tests, electrolyte panel, coagulation profile, and serum amylase levels were all within normal ranges (Table 1).

**Table 1:** Patient's vital signs and selected laboratory test results before and after the procedure (six hours post-TACE), along with their reference ranges. TACE: transarterial chemoembolization

Parameter	Preoperative	Postoperative	Reference range (unit)
Body temperature (BT)	36.5℃	38.3℃	36-37.3 (℃)
Heart rate (HR)	76	122	60-100 (beats/min)
Blood pressure (BP)	124/76	144/85	90-130/60-90 (mmHg)
Peripheral oxygen saturation (SpO₂)	100%	92%	95-100 (%)
White blood cell count (WBC)	9.23	15.6	4-10 x 10⁹/L
Neutrophil count (NEUT)	6.44	12.5	2-6 x 10⁹/L
Hemoglobin (Hb)	132	135	130-160 (g/L）
C-reactive protein (CRP)	2.5	185	＜5 (mg/L)
Serum amylase (AMY)	32	535	＜40 (U/L)
Alpha-fetoprotein (AFP)	986.8	950.2	＜10 (ng/mL)
Alanine aminotransferase (ALT)	40	112	＜40 (U/L)
Aspartate aminotransferase (AST)	32	97	＜30 (U/L)
Total bilirubin (TBIL)	15	27	＜17 (μmol/L)
Direct bilirubin (DBIL)	9	18	＜10 μmol/L
Lipase (LIP)	17	98	＜10 (U/L)

The patient's liver function was classified as Child-Pugh A. The patient opted for TACE as treatment. During the procedure, following standard antiseptic preparation and draping, a 5F sheath was introduced via femoral artery access. An RH catheter was selectively engaged in the celiac trunk. Angiography revealed tortuous hepatic arteries with abundant pathological vasculature, without evidence of aneurysms or early portal vein opacification (Figure 1D). Angiographic analysis revealed a distinctive anatomical predisposition: the common hepatic artery gave rise to the proper hepatic artery and a large pancreatoduodenal arcade branch at an almost 180° horizontal angle. A microcatheter was then advanced superselectively into the branch of the right hepatic artery supplying the dominant tumor (Figure 1E).

During infusion of an embolic mixture containing 5 g of Lipiodol and drug-eluting microspheres loaded with epirubicin (total dose: 75 mg), the patient experienced an abrupt onset of severe upper abdominal pain, accompanied by tachycardia (heart rate 120 bpm) and significant agitation. Control angiography demonstrated markedly slowed flow in the embolized hepatic arterial branch with signs of reflux, along with lipiodol deposition in the liver tumor, leading to procedure termination (Figure 1F). The definitive opacification of the pancreatoduodenal arcade with embolic material was not directly visualized.

Postoperatively, the patient experienced persistent, significant abdominal pain. An emergency non-contrast abdominal CT scan showed that the previously noted hyperdense nodule in the gallbladder neck remained unchanged (Figure 1G). At the same time, the pancreatic head appeared swollen with surrounding streaky infiltrates, fluid collections, and thickening of the anterior renal fascia (Figure 1H) (six hours post-TACE). Subsequent laboratory tests revealed an elevated white blood cell count of 15.6 × 10⁹/L with neutrophilic predominance (12.5 × 10⁹/L), a significantly increased serum amylase level of 535 U/L, a markedly elevated serum lipase level of 320 U/L, and a markedly elevated C-reactive protein level of 185 mg/L, confirming the diagnosis of acute pancreatitis (six hours post-TACE).

The diagnosis of acute pancreatitis was confirmed using established clinical criteria, which require meeting at least two of the following three features: (1) abdominal pain consistent with the disease, (2) serum amylase and/or lipase levels >3 times the upper limit of normal, and (3) characteristic findings on cross-sectional imaging. Our patient met all three criteria, with acute abdominal pain, a serum amylase level of 535 U/L (>3 times the upper limit of 40 U/L), and CT evidence of peripancreatic inflammation. The patient immediately received aggressive intravenous fluid resuscitation, analgesic support, and standard medical therapy for acute pancreatitis. Based on the Revised Atlanta Classification, the acute pancreatitis was graded as moderately severe. This determination was made because the patient exhibited transient organ failure (evidenced by tachycardia and hypoxemia), which resolved within 48 hours of onset with appropriate medical management. His symptoms gradually improved, and he was discharged after one week of hospitalization.

A three-month follow-up CT scan showed a partial response according to mRECIST criteria, with a reduction in size and contrast enhancement of hepatic tumors. Peripancreatic inflammatory changes had resolved, though some nodular and patchy low-density shadows remained in the pancreatic border and adjacent mesentery region (Figure 1I).

## Discussion

The severe acute abdominal pain occurring during TACE in this patient requires a thorough etiological analysis. While mild to moderate abdominal pain, fever, and nausea are common manifestations of PES and are typically self-limiting, their severity in our case, manifesting as an acute abdominal crisis with hemodynamic changes, was disproportionate to what is characteristically expected from PES alone. This marked discrepancy in clinical presentation was a pivotal factor in prompting the search for an alternative, more serious etiology such as acute pancreatitis [3]. Postoperative imaging and laboratory findings clearly indicated acute pancreatitis, providing a reasonable explanation for the patient's dramatic clinical presentation. However, the specific etiology of pancreatitis in this context requires careful differential diagnosis.

First, the presence of a 0.6 cm stone in the gallbladder neck raised the possibility of gallstone-induced pancreatitis. The patient did exhibit elevations in liver enzymes (ALT, AST) and bilirubin following the TACE procedure; however, these are known, common sequelae of the chemoembolization itself. To definitively rule out a biliary etiology for the concurrent pancreatitis, we relied on cross-sectional imaging. Serial abdominal CT scans confirmed that the gallstone remained lodged in the gallbladder neck without passage into the cystic or common bile duct. Most conclusively, a magnetic resonance cholangiopancreatography was performed, which demonstrated a patent biliary system without any evidence of ductal dilation or obstruction. This combination of findings securely excludes a biliary cause for the acute pancreatitis.

Second, although chemotherapeutic drugs used in TACE (such as epirubicin) have been rarely reported to cause drug-induced pancreatitis, the virtually instantaneous onset of symptoms following drug infusion in this case, within approximately one minute, does not conform to the characteristics of typical drug reactions.

This mechanism, while not directly proven by angiographic observation of reflux, is the most consistent with the totality of the evidence. The immediate symptom onset during embolization is a critical piece of circumstantial evidence that strongly suggests a procedural cause. Furthermore, the patient's vascular anatomy presented a known risk factor for reflux. It is important to note that the drug-eluting microspheres themselves are not radiopaque, meaning that their reflux could occur without a clear fluoroscopic signature. Thus, the diagnosis of pancreatitis induced by embolization remains a clinical one, based on the exclusion of alternatives and the compelling temporal relationship.

We hypothesize that during injection of the lipiodol and epirubicin-loaded microsphere mixture, factors such as injection pressure, hepatic artery hemodynamics, or increased vascular resistance due to underlying cirrhosis may have caused partial reflux of embolic material into the pancreatoduodenal arteries. The pancreas, particularly the head region, depends on terminal artery supply and is highly sensitive to ischemia. Embolic particle occlusion of pancreatic microcirculation can initiate local ischemic injury, triggering a cascade of intracellular lysosomal enzyme activation and abnormal zymogen activation within pancreatic cells, ultimately leading to pancreatic autodigestion and the development of clinical acute pancreatitis. This ischemic mechanism aligns well with literature reports and satisfactorily explains the clinical presentation and timeline in this case [4,6].

TACE remains a crucial intervention for unresectable HCC, serving as a bridge to transplantation or downstaging therapy [2]. A review of the outcomes in Table 2 reveals that post-TACE pancreatitis carries a variable but non-negligible risk of severe morbidity and mortality. The clinical course of our patient, which was characterized as moderately severe, occupies a middle ground in this spectrum. This comparison reinforces that the management of this complication is not uniform but must be stratified according to its initial severity, with our case serving as an example of a severe presentation that was successfully reversed through vigilant supportive management [5]. We searched PubMed and Google Scholar databases for articles published up to September 30, 2025, using the following key terms: "TACE", "transarterial chemoembolization", "acute pancreatitis", "complication", and "hepatocellular carcinoma". According to our review of literature on TACE-induced AP (Table 2), the reported incidence of clinically apparent AP post-TACE ranges from 1.7% to 2% [4-10]. However, the true incidence may be significantly higher when including subclinical pancreatitis, which is manifested only by enzyme elevation.

**Table 2:** Research publications on TACE complicated by pancreatitis A total of seven publications are included, comprising five case reports and two retrospective analyses. The literature summary presented in this case report is based on a narrative review. We searched PubMed and Google Scholar databases for articles published up to September 30, 2025, using the following key terms: "TACE", "transarterial chemoembolization", "acute pancreatitis", "complication", and "hepatocellular carcinoma". This search was conducted to provide a clinical context for our case and was not intended as a systematic review. TACE: transarterial chemoembolization

Author/year	Number	Prognosis
Gjoreski et al. (2019) [4]	Twenty subjects with 29 tumours were treated. Three patients had insignificant ascites. One patient had a procedure-related SAE (acute pancreatitis) within the postembolization period, which was induced. Six-month freedom from procedure-related SAE or death was 95% (one necrotizing pancreatitis).	Not available
Ozçinar et al. (2009) [5]	Case (1)	Remission
Krishnamurthy et al. (2017) [6]	Case (1)	Remission
Marcacuzco Quinto et al. (2018) [7]	Out of the 196 patients: acute cholecystitis (4), acute pancreatitis (3), liver rupture (1), liver abscess (1), and renal failure (1).	Not available
Addario et al. (2008) [8]	Case (1)	Remission
Chey et al. (2006) [9]	Case (1)	Remission
Yamaguchi et al. (2018) [10]	case(1)	Death

Multiple risk factors contribute to AP following TACE. Beyond the established roles of embolic microsphere volume and specific chemotherapeutic agents (such as carboplatin) as primary risk factors [7], vascular anatomical variations deserve consideration. Although some studies haven't confirmed strong associations with AP [8], certain specific variants, such as the right hepatic artery originating from the superior mesenteric artery, may alter local hemodynamics and increase the risk of embolic material reflux [9]. Additionally, the characteristics of embolic agents represent contributing factors; for instance, compared to lipiodol, newer microspheres (e.g., DC Beads) exhibit poorer radiopacity under fluoroscopy, making intraprocedural reflux monitoring more challenging [10].

In conclusion, our recommendations for preventing this complication can be stratified by their evidence base. Well-established, evidence-based measures include superselective catheterization and meticulous fluoroscopic monitoring to avoid reflux, which are supported by procedural guidelines and technical notes [3]. Based on the mechanism observed in our case, we further suggest that particular caution should be exercised in patients with variant anatomy, like the wide-origin pancreatoduodenal artery seen here. Furthermore, clinicians should be aware that the inflammatory sequelae of non-target embolization may be exacerbated by the sustained release of chemotherapeutic agents from modern drug-eluting embolics. This distinction highlights core procedural standards while sharing experiential insights that may guide management in complex scenarios. Therefore, operators should position catheter tips as distally as possible in the target arteries, closely monitor the embolization process under fluoroscopy, strictly control injection pressure and embolic dose, and adjust accordingly to minimize reflux-related non-target embolization.

Diagnosing AP following TACE presents significant challenges due to symptom overlap with common PES, frequently leading to delayed diagnosis. In this case, the patient's severe and worsening pain was initially misinterpreted as part of a self-limiting PES. This highlights a common diagnostic pitfall: even severe, progressive symptoms can be mistakenly attributed to benign causes. Consequently, this experience underscores that any severe or persistent pain post-TACE warrants immediate investigation to rule out catastrophic complications like acute pancreatitis. Based on the clinical logic derived from documented cases of delayed presentation [8,10], we recommend routine dynamic monitoring of serum amylase and lipase in patients with significant post-TACE abdominal pain. While no formal guideline exists for this specific monitoring protocol, this approach is a prudent clinical response to the recognized risk and variable timing of this complication.

## Conclusions

This case provides a detailed account of the diagnosis and management of acute pancreatitis complicating TACE in an HCC patient. Through supportive care targeting the presumed ischemic injury from non-target embolization, the patient's symptoms resolved, and he was successfully discharged. By excluding common causes such as biliary and drug-induced etiologies, we have inferred that the causative mechanism is pancreatic ischemic injury resulting from the reflux of embolic material. This conclusion, while not directly proven, is the most consistent with the immediate onset of symptoms during the embolic infusion and the supportive imaging findings. This mechanism alerts interventional operators that even during apparently smooth embolization procedures, subtle hemodynamic changes can lead to serious non-target embolization events.

The diagnostic process in this case highlights the challenges in differentiating post-TACE AP. The initial presentation overlapped with PES, but the disproportionate severity of the abdominal pain and its persistence beyond 48 hours raised the first red flag. The definitive differentiation, however, came from the biochemical and radiological workup: a marked elevation of both amylase (535 U/L) and lipase (320 U/L), coupled with CT evidence of peripancreatic fat stranding and fluid collections. This triad of severe pain, significant enzymemia, and confirmatory imaging is what ultimately secured the diagnosis. We strongly advocate for routine postoperative serum amylase and lipase monitoring in patients with significant pain, as they serve as critical objective biomarkers to guide this essential differential diagnosis. We strongly advocate for routine postoperative serum amylase and lipase monitoring in patients with significant abdominal pain as essential for early detection, avoiding misdiagnosis, and treatment delays.

Prevention remains paramount in managing TACE-related pancreatitis. This requires operators to consistently adhere to superselective catheterization, slow and careful injection under fluoroscopic guidance, and close monitoring of intravascular flow status to prevent reflux. Reflecting on our case, two factors made complete prevention challenging: the high-risk anatomy predisposing to reflux and the limitations of fluoroscopy in visualizing microsphere distribution. This illustrates that standard preventive strategies, while essential, must be supplemented with heightened caution in patients with unfavorable anatomy and an awareness of the limitations of our imaging tools. This case underscores the importance of maintaining constant vigilance, as complications can still arise even when procedural guidelines are strictly followed. This case offers valuable experience for interventional radiologists and hepatologists, emphasizing that while pursuing tumor treatment efficacy, constant vigilance and active prevention of potentially life-threatening complications are imperative.
